# Perioperative Rhabdomyolysis Following Prolonged Laparoscopic Radical Nephrectomy in the Lateral Decubitus Position

**DOI:** 10.1002/ccr3.73640

**Published:** 2026-09-28

**Authors:** Madeline Dow, Aariya Srinivasan, Sabry Ayad

**Affiliations:** ^1^ Department of Anesthesiology Cleveland Clinic Foundation Ohio USA; ^2^ Department of Anesthesiology Outcomes Research Consortium USA; ^3^ Staff Anesthesiologist, Cleveland Clinic, Fairview Hospital Ohio USA

**Keywords:** acute care, anesthesia, critical care medicine, urology/andrology

## Abstract

Perioperative rhabdomyolysis can occur after prolonged laparoscopic nephrectomy, particularly in obese patients positioned in the lateral decubitus position. In high‐risk patients undergoing prolonged surgery, early postoperative creatine kinase measurement is essential for timely diagnosis. Prompt hydration and renal monitoring are critical to prevent acute kidney injury and optimize patient outcomes.

## 1 Introduction

Rhabdomyolysis is characterized by muscle breakdown with release of intracellular constituents, including myoglobin, potassium, phosphate, and creatine kinase (CK) into the circulation [1, 2]. Disruption of the myocyte membrane leads to calcium influx and ATP depletion, resulting in irreversible muscle fiber necrosis [1, 2]. These shifts can lead to hyperkalemia, hyperphosphatemia, and hypocalcemia. They may also result in rebound hypercalcemia in some cases during the recovery phase [2]. The resulting myoglobinuria places patients at risk for acute kidney injury and systemic electrolyte disturbances. Although the major factors associated with rhabdomyolysis are traumatic injuries and prolonged immobilization, increasing evidence indicates that intraoperative factors such as patient positioning and surgical duration can cause significant muscle injury [3]. We report a case of perioperative rhabdomyolysis in a 53‐year‐old man with Class II obesity after a prolonged robotic laparoscopic radical left nephrectomy performed in the right flank position.

## 2 Case Presentation/Examination

A 53‐year‐old man was admitted for a scheduled robotic laparoscopic radical left nephrectomy with para‐aortic and interaortocaval lymph node dissection to resect a left renal neoplasm with renal pelvis lymph node enlargement. The patient was 188 cm tall and weighed 136 kg, corresponding to an estimated body mass index (BMI) of 38.5 kg/m^2^. His past medical history included Type 2 diabetes mellitus, Class II obesity, hypertension, and nicotine use disorder with a 36‐pack‐year history of tobacco use. The patient had an American Society of Anesthesiologists (ASA) physical status of 3.

## 3 Methods

Cefazolin 3 g was administered intravenously prior to induction of anesthesia in accordance with institutional weight‐based dosing protocols. General anesthesia was induced with 2 mg midazolam, 100 mg lidocaine, 250 mg propofol, and 100 mg rocuronium. Following induction of general anesthesia, the patient was positioned in the right flank position with pressure‐relieving padding applied to joints and pressure points. Pressure points were reassessed approximately every 15 min throughout the procedure. The intubation, line placement, and anesthetic course were uneventful. The patient was given 3 L of lactated Ringer's solution. A total of 750 mL of 5% albumin was administered for intravascular volume expansion to support hemodynamic stability during the prolonged procedure. A phenylephrine infusion at 25 μg/min was also administered for blood pressure support. The kidney was successfully extracted with no intraoperative bleeding from the nephrectomy site. No intraoperative repositioning was performed. The total estimated blood loss was 250 mL, intraoperative urine output was 115 mL, and the total operative time was 5 h and 15 min.

Immediately upon emergence from anesthesia, the patient appeared agitated and complained of pain in the right buttock despite 1.5 mg of hydromorphone and continuation of dexmedetomidine infusion for a total of 164.85 μg. He reported no pain along the left flank. CT imaging revealed an enlarged right gluteus medius consistent with an intramuscular hematoma. Fluid and stranding tracked into the right posterior thigh deep to the gluteus maximus and anterior to the hamstring musculature. In the right upper outer gluteal quadrant, compartment pressure measured with a Stryker needle was mildly elevated at 12 mmHg. An ultrasound performed immediately following the CT imaging did not reveal a hematoma or fluid collection. Given the patient's severe right gluteal pain and concern for underlying muscle injury, laboratory evaluation was undertaken.

Initial laboratory studies obtained on postoperative day (POD) 1 revealed a markedly elevated serum CK of 53,067 U/L (reference 51–298 U/L), normal creatinine of 1.19 mg/dL (reference 0.73–1.22 mg/dL), calcium of 8.7 mg/dL (reference 8.5–10.2 mg/dL), and elevated serum myoglobin of 1625 ng/mL (reference < 91 ng/mL). Rhabdomyolysis was diagnosed based on the patient's acute postoperative gluteal pain, markedly elevated serum CK level, and elevated serum myoglobin. Review of the anesthesia record did not reveal episodes of prolonged or untreated intraoperative hypotension, and hypotension was not considered a significant contributor to the rhabdomyolysis in this case.

Following diagnosis of rhabdomyolysis on POD 1, aggressive intravenous hydration was initiated with 100 mEq sodium bicarbonate in 1 L of 0.45% sodium chloride. The bicarbonate‐containing fluid was prescribed in accordance with institutional practice with the goal of urinary alkalinization and was continued at a documented rate of 200 mL/h through POD 2. A Foley catheter urinalysis on POD 1 showed a urine pH of 6.5. During the 24‐h period ending on the morning of POD 2, the patient had 2209 mL of recorded intake and 1900 mL of recorded output.

Serial CK monitoring was performed in accordance with institutional practice and demonstrated progressive improvement in CK and renal function after initiation of hydration. CK decreased to 17,712 U/L later on POD 1 and to 11,011 U/L on POD 2. Creatinine transiently increased to 1.38 mg/dL later on POD 1 and improved to 1.18 mg/dL on POD 2. Calcium was 8.0 mg/dL on POD 2. The patient's activity level gradually increased, and pain was controlled on oral medications. Physical examination showed mild bulging in the left lower quadrant at the nephrectomy site, consistent with a postoperative surgical‐site finding unrelated to the right gluteal pathology, with well‐healed surgical incisions and mild bilateral lower extremity edema. He was discharged on POD 3.

## 4 Conclusion and Results

Upon follow‐up on POD 13, the patient was referred to oncology for consideration of adjuvant pembrolizumab to address the increased risk of recurrence for clear‐cell renal‐cell carcinoma after surgery. An ultrasound of the abdominal wall was scheduled to evaluate for fluid collection. He was prescribed oxycodone‐acetaminophen (5 mg/325 mg) for the reported pain in his wound and right buttock. As part of institutional follow‐up protocol after rhabdomyolysis, serum CK was remeasured at one month. The serum CK level was 72 U/L, creatinine was 1.27 mg/dL, and calcium was 10.9 mg/dL.

## 5 Discussion

Perioperative rhabdomyolysis can be caused by patient‐related and procedural factors that predispose skeletal muscle to ischemia and reperfusion injury. It is a rare complication of urologic surgery, with a reported incidence of approximately 0.1% in a large national cohort study [4]. Established risk factors include obesity, male sex, prolonged operative times, and lateral decubitus positioning, which have been consistently identified in reports of laparoscopic renal surgery [5]. In the present case, an elevated BMI of 38.5 kg/m^2^ and prolonged right flank positioning may have resulted in sustained compression of the dependent gluteal musculature across a 5‐h operating period, potentially contributing to the marked elevations in CK and serum myoglobin. CT demonstrated enlargement of the right gluteus medius with associated fluid and stranding, interpreted as likely intramuscular hematoma, whereas subsequent ultrasound demonstrated no mass or fluid collection. Although the imaging findings were discordant, the working clinical interpretation remained a suspected right gluteal hematoma, consistent with the CT findings, as reflected in subsequent clinical documentation. However, this CT abnormality and the biochemical evidence of rhabdomyolysis may not have represented the same pathological process. Nevertheless, the overall clinical presentation, including acute postoperative gluteal pain following prolonged surgery and markedly elevated CK and serum myoglobin, favored perioperative rhabdomyolysis as the principal diagnosis. The exact timing and mechanism of the suspected right gluteal hematoma could not be determined from the available perioperative documentation. No specific intraoperative event was identified that clearly accounted for its development.

A similar case demonstrated that prolonged laparoscopic nephrectomy in the fully flexed flank position can increase pressure on the dependent gluteal muscles and contribute to rhabdomyolysis [6]. A three‐hour operative duration has been identified as a critical threshold for risk of rhabdomyolysis in obese patients undergoing laparoscopic nephrectomy [7]. For such cases, early serum CK measurement is recommended on the day of surgery for timely recognition and management of rhabdomyolysis [7]. The patient's five‐hour procedure exceeded this threshold, underscoring the importance of intraoperative risk identification and postoperative monitoring.

Serum CK is a sensitive biomarker of muscle injury, with levels correlating closely with symptom severity [7]. Higher peak CK levels have been associated with an increased risk of acute kidney injury, while aggressive hydration may reduce this risk [4]. In our case, CK concentrations of over 50,000 U/L prompted immediate hydration to maintain urine output and prevent nephrotoxicity. The patient's peak CK level of 53,067 U/L on POD 1 was substantially above commonly used thresholds for clinically significant rhabdomyolysis. Prior reviews have noted increased risk of kidney injury at CK levels greater than 5000 U/L, and CK levels greater than 15,000 U/L have been classified as severe rhabdomyolysis [1, 2, 3]. This value is comparable to a previously reported case of rhabdomyolysis after prolonged laparoscopic radical nephrectomy, in which the POD 1 CK level was 51,050 U/L [6].

Once muscle injury occurs, fluid shifts from the intravascular space into damaged muscle tissue. The resulting “third space” contributes to hypovolemia and renal ischemia [8]. Myoglobin released from injured muscle is filtered by the kidneys and can cause tubular obstruction, direct tubular toxicity, and renal vasoconstriction, increasing the risk of acute kidney injury [2, 8]. Early volume resuscitation helps preserve renal perfusion and reduce myoglobin‐mediated renal injury. Hypovolemia further limits renal blood flow through sympathetic activation, vasoconstrictor release, and nitric oxide degradation [8]. At the same time, injured myocytes sequester calcium, often leading to early hypocalcemia. However, replacing calcium is avoided unless there is concurrent hyperkalemia with ECG changes or symptoms of hypocalcemia due to its high intracellular concentration [8]. During recovery, calcium may be released back into circulation, causing rebound hypercalcemia.

The role of adjunctive therapies such as mannitol and sodium bicarbonate in rhabdomyolysis remains controversial. Mannitol and sodium bicarbonate were previously used in the management of rhabdomyolysis. Mannitol was used to promote osmotic diuresis, while bicarbonate was used to alkalinize the urine [3]. However, more recent evidence does not support their routine use to prevent acute kidney injury. Current management instead emphasizes early intravascular volume expansion and avoidance of agents that decrease renal blood flow [9]. Notably, although CK and serum myoglobin levels were elevated, renal replacement therapy was not required in this case. Other reported cases have required hemodialysis despite aggressive hydration [5], underscoring the importance of early recognition and management.

Preventive strategies include optimizing pressure distribution with padding, minimizing flank flexion, and periodically repositioning the patient during surgery [10]. Postoperative monitoring of CK and renal function enables early detection of muscle injury [7]. These measures may help reduce the risk of rhabdomyolysis and its associated complications during radical laparoscopic nephrectomy.

## Author Contributions

**Madeline Dow:** conceptualization, visualization, writing – original draft, writing – review and editing. **Aariya Srinivasan:** conceptualization, visualization, writing – original draft, writing – review and editing. **Sabry Ayad:** conceptualization, supervision, visualization, writing – review and editing.

## Funding

The authors have nothing to report.

## Consent

Written informed consent was acquired from the patient to publish this report in accordance with the journal's patient consent policy.

## Conflicts of Interest

The authors declare no conflicts of interest.

## Data Availability

The data supporting this report's findings are available on request from the corresponding author. The data are not publicly available due to privacy or ethical restrictions.
